# Pathology of experimentally induced mouthrot caused by *Tenacibaculum maritimum* in Atlantic salmon smolts

**DOI:** 10.1371/journal.pone.0206951

**Published:** 2018-11-01

**Authors:** Kathleen Frisch, Sverre Bang Småge, Renate Johansen, Henrik Duesund, Øyvind Jakobsen Brevik, Are Nylund

**Affiliations:** 1 Cermaq Group AS, Oslo, Norway; 2 Fish Disease Research Group, Department of Biology, University of Bergen, Bergen, Norway; 3 PHARMAQ Analytiq AS, Oslo, Norway; INRA, FRANCE

## Abstract

Mouthrot, caused by *Tenacibaculum maritimum* is a significant disease of farmed Atlantic salmon, *Salmo salar* on the West Coast of North America. Smolts recently transferred into saltwater are the most susceptible and affected fish die with little internal or external clinical signs other than the characteristic small (usually < 5 mm) yellow plaques that are present inside the mouth. The mechanism by which these smolts die is unknown. This study investigated the microscopic pathology (histology and scanning electron microscopy) of bath infected smolts with Western Canadian *T*. *maritimum* isolates TmarCan15-1, TmarCan16-1 and TmarCan16-5 and compared the findings to what is seen in a natural outbreak of mouthrot. A real-time RT-PCR assay based on the outer membrane protein A specific for *T*. *maritimum* was designed and used to investigate the tissue tropism of the bacteria. The results from this showed that *T*. *maritimum* is detectable internally by real-time RT-PCR. This combined with the fact that the bacteria can be isolated from the kidney suggests that *T*. *maritimum* becomes systemic. The pathology in the infected smolts is primarily mouth lesions, including damaged tissues surrounding the teeth; the disease is similar to periodontal disease in mammals. The pathological changes are focal, severe, and occur very rapidly with little associated inflammation. Skin lesions are more common in experimentally infected smolts than in natural outbreaks, but this could be an artefact of the challenge dose, handling and tank used during the experiments.

## Introduction

Tenacibaculosis is a disease characterized by frayed fins, tail rot, mouth erosion, and skin lesions that are often ulcerative; it causes significant losses in a number of economically important marine fish species worldwide [1,2]. Three species belonging to the genus *Tenacibaculum* have been associated with this clinical presentation in farmed Atlantic salmon (*Salmo salar*): *Tenacibaculum dicentrarchi* [3], "*Tenacibaculum finnmarkense*" [4–6], and *Tenacibaculum maritimum* [7]. However, the clinical presentation of *T*. *maritimum* infections in Atlantic salmon smolts in the Pacific Northwest (British Columbia (BC), Canada and Washington, USA) is different from classical tenacibaculosis (as described above) and is commonly referred to as mouthrot [8–11]. Cultured Pacific salmon species (e.g. Chinook salmon, *Oncorhynchus tshawytscha*) in the Pacific Northwest appear to be resistant to developing mouthrot [9].

Mouthrot typically affects smolts recently transferred into saltwater, and has been present in the Pacific Northwest since the late 80s [12]. Due to a lack of preventative measures against this disease, mouthrot continues to be the main reason that antibiotics are used in the production of Atlantic salmon in the region [13]. Mouthrot is generally diagnosed by the presence of distinctive yellow plaques associated primarily with the teeth of affected smolts [10,14]. This clinical manifestation of *T*. *maritimum* infections has not been reported in any other Atlantic salmon farming region even in areas where *T*. *maritimum* is present.

The pathology of mouthrot in the Pacific Northwest was first described in the early 90s, before the bacterial agent was identified [12,14]. Gross pathology includes focal yellow bacterial mats around the palate and teeth. The lesions range from small and hardly visible to multiple with erosion of the upper and/or lower jaw in severe cases [14]. Microscopic examination of these lesions were described as “mats of *Cytophaga*-like filamentous bacteria associated with areas of ulceration and necrosis often extending into the underlying bone" [12]. Major taxonomical revisions have since identified these “*Cytophaga*-like” bacteria as *T*. *maritimum* [15,16]. Diseased individuals die with little or no other gross external or internal lesions other than these typical “yellow plaques” in the mouth, and there is no evidence of concurrent disease [10].

When Atlantic salmon smolts are experimentally bath infected with one high dose of Western Canadian *T*. *maritimum*, clinical signs are not exclusive to the mouth; the gills and skin can also be affected [11]. Necrotic gill lesions have sometimes been observed in mouthrot affected smolts in BC (personal observations, Frisch); however, this is not a common finding. Gill lesions associated with this bacterium have also been noted in naturally and experimentally infected Atlantic salmon smolts in Tasmania [7,17] and Chinook salmon in California [18]. Skin lesions are also more common in experimentally infected smolts than in natural outbreaks, but this could be an artefact of the experiments [11].

The mechanism by which *T*. *maritimum* kills Atlantic salmon smolts in the Pacific Northwest while only causing very small mouth lesions continues to be a mystery. This study describes for the first time the pathology associated with experimentally induced mouthrot and compares it to what is normally seen in natural outbreaks of this disease. Tissue tropism of the bacteria, using the newly developed real-time RT-PCR is also investigated.

## Materials and methods

### Real-time RT-PCR for *T*. *maritimum*

Prior to this publication, there was only one published real-time RT-PCR assay specific for *T*. *maritimum* [19]. The assay targets the 16S rRNA gene and was tested using DNA [19]. However, the 16S rRNA gene has low phylogenic resolution at the species level when compared to other genes [20], and real-time RT-PCR assays based on this gene may not be very specific. The new real-time RT-PCR assay (Tmar_ompA) targets the outer membrane protein A (*ompA*) gene (forward primer: GCCAATAGCAACGGGATACC, reverse primer: TCGTGCGACCATCTTTGGT, probe: TGAATCAAATGCGATCTT). An alignment of the *ompA* gene using available *Tenacibaculum* spp. sequences in the GenBank and from the *T*. *maritimum* strains TmarCan15-1, TmarCan16-1, TmarCan16-5, NLF-15, and Ch-2402 [16,21,22] (also available in the GenBank) was used during the design of the assay.

The specificity of Tmar_ompA, based on this alignment, was tested using RNA extracted from clonal cultures of *Tenacibaculum* spp. The aim for the assay was to amplify *T*. *maritimum* strains NCIMB 2154^T^, TmarCan15-1, TmarCan16-1, TmarCan16-5, NLF-15, and Ch-2402 [16,21,22], and not to amplify *Tenacibaculum adriaticum* DSM18961^T^, *Tenacibaculum dicentrarchi* USC35/09^T^, "*Tenacibaculum finnmarkense*" HFJ^T^, *Tenacibaculum ovolyticum* EKD-002^T^ and *Tenacibaculum soleae* LL0412.1.7^T^. To compare this new assay to the already published one, these RNA samples were also tested using the assay developed by Fringuelli, Savage [19]. Tmar_ompA was optimized and the efficiency determined using 10-fold dilutions of RNA extracted from TmarCan15-1 [16] and from known positive skin tissue samples from the cohabitation experiment described in Frisch, Småge [11].

All RNA was extracted using TRI Reagent (Sigma-Aldrich) following the manufacturer’s protocol, except that an additional washing step using 100% ethanol was performed prior to air drying the RNA pellet. Extracted RNA was stored at -80°C. All assays were run using an AgPath-ID kit (Thermo Fisher Scientific) with 2 μL of RNA and the standard concentrations of primers (400 nM) and probe (120 nM). Each run consisted of 45 cycles.

### Cohabitation experiment

Tissue samples from a previously published cohabitation experiment [11] were used to investigate the tissue tropism of the bacteria through real-time RT-PCR screening. In this experiment six groups of 20 Atlantic salmon smolts (shedders) were bath infected with three different isolates of *T*. *maritimum* (TmarCan15-1, TmarCan16-1 and TmarCan16-5) that came from natural mouthrot outbreaks on BC Atlantic salmon farms [16]. The shedders were bath infected for 5 hours in 12°C saltwater (34 ppt) using one of the above isolates (groups 4–1 and 4–2 with 1.68 x 10^7^ cells mL^-1^ TmarCan15-1, groups 4–3 and 4–4 with 1.78 x 10^7^ cells mL^-1^ TmarCan16-5 and groups 4–5 and 4–6 with 8.75 x 10^5^ cells mL^-1^ TmarCan16-1). Two additional groups of 20 shedders were used as controls (4–7 and 4–8), one bath exposed to 1 L marine broth (Difco 2216) (MB) and the other untouched. 24 hours post-bath infection, 40 smolts (cohabitants) were added to each group. The husbandry conditions are described in Frisch, Småge [11] and results are summarized in Table 1. The mouth, gill and skin lesions visible macroscopically on mortality were scored as described in Frisch, Småge [11] and are summarized in Table 2.

**Table 1:** Cohabitation experiment groups.

Group	Number of Fish	Isolate	Bacterial Bath Concentration (cells mL^-1^)	Accumulated Percent Mortality	Start of Mortality (days post-exposure)	End of Mortality (days post-exposure)
4–1	20 shed 40 cohab	TmarCan15-1	1.68 x 10^7^	shed: 100 cohab: 75	shed: 2 cohab: 9	shed: 7 cohab: 20
4–2	20 shed 40 cohab	TmarCan15-1	1.68 x 10^7^	shed: 100 cohab: 76	shed: 3 cohab: 7	shed: 7 cohab: 17
4–3	20 shed 40 cohab	TmarCan16-5	1.78 x 10^7^	shed: 95 cohab: 27	shed: 2 cohab: 12	shed: 16 cohab: 17
4–4	20 shed 40 cohab	TmarCan16-5	1.78 x 10^7^	shed: 84 cohab: 31	shed: 3 cohab: 10	shed: 10 cohab: 20
4–5	20 shed 40 cohab	TmarCan16-1	8.75 x 10^5^	shed: 100 cohab: 100	shed: 3 cohab: 6	shed: 5 cohab: 11
4–6	20 shed 40 cohab	TmarCan16-1	8.75 x 10^5^	shed: 100 cohab: 100	shed: 3 cohab: 6	shed: 6 cohab: 9
4–7	20 shed 40 cohab	Control (Marine Broth)	1 L	shed: 0 cohab: 0	-	-
4–8	20 shed 40 cohab	Control (no exposure)	N/A	shed: 0 cohab: 0	-	-

This table is a summary of the group descriptions and results from the cohabitation experiment in Frisch, Småge [11] (shed refers to shedders and cohab refers to cohabitants). The isolates used were collected from natural outbreaks of mouthrot on Atlantic salmon farms in BC, Canada [16]. Accumulated percent mortality is shown for each group, as well as the time period post-exposure that mortality occurred. In general, the mortality curve for each group had a sigmoid shape.

**Table 2:** Cohabitation experiment gross lesion scoring of mortality.

Tissue	Score	Shedders (% of total mortality)			Cohabitants (% of total mortality)		
		TmarCan15-1	TmarCan16-1	TmarCan16-5	TmarCan15-1	TmarCan16-1	TmarCan16-5
Mouth	0	62.5	94.9	27.8	-	82.7	-
	1	30.0	5.1	19.4	13.0	13.6	19.0
	2	2.5	-	33.3	35.2	3.7	57.1
	3	5.0	-	19.4	51.9	-	23.8
Skin	0	47.5	97.4	36.1	9.3	88.9	28.6
	1	45.0	2.6	22.2	42.6	11.1	28.6
	2	5.0	-	33.3	33.3	-	33.3
	3	2.5	-	8.3	14.8	-	9.5
Gills	0	32.5	100.0	88.9	94.4	100.0	95.2
	1	35.0	-	5.6	5.6	-	4.8
	2	32.5	-	5.6	-	-	-

Scoring of external clinical signs seen in mortality in the cohabitation experiment as a percentage of total mortality. Duplicate groups are combined. Scores were 0 to 3 for mouth and skin lesions, and 0 to 2 for gill lesions as described in Frisch, Småge [11], with 0 being no visible abnormalities and 2 or 3, the most severe.

The cohabitation experiment was approved by the Norwegian Food Safety Authority (Mattilsynet) under the identification code 16/207694.

### Cohabitation experiment tissue screening

The mouth and gills of five diseased cohabitants from each group were sampled with the exception of the 2 control groups that had no mortality. The brain, heart, kidney and skin mucus were also sampled from two smolts of each of these groups. At days 7 and 14 post-infection, two randomly selected apparently healthy cohabitants were sampled (mouth, gills, brain, heart, kidney and skin mucus) from each group. However, due to the rapid mortality in groups 4–5 and 4–6, this was not possible in these groups. The day 7 samples in group 4–2 were also missed. All samples were collected aseptically and kept on ice and then stored at -20°C. Moribund smolts and randomly selected cohabitants were euthanized with a swift blow to the head.

RNA was extracted from each of these samples and screened using the Tmar_ompA assay using the above protocol. An assay targeting the elongation factor 1 alpha (EF1A) was used on the mouth, gills, brain, heart and kidney samples as an endogenous control (forward primer: CCCCTCCAGGACGTTTACAAA, reverse primer: CACACGGCCCACAGGTACA, probe: ATCGGTGGTATTGGAAC) [23]. Due to the variability of an endogenous control such as EF1A in skin mucus, these samples were spiked with cultured *Halobacterium salinarum* DSM 3754^T^ cells suspended in PBS prior to the RNA extraction. This exogenous control was detected using the Sal assay (forward primer: GGGAAATCTGTCCGCTTAACG, reverse primer: CCGGTCCCAAGCTGAACA, probe: AGGCGTCCAGCGGA) [24].

### Microscopic pathology

**Fig 1:**
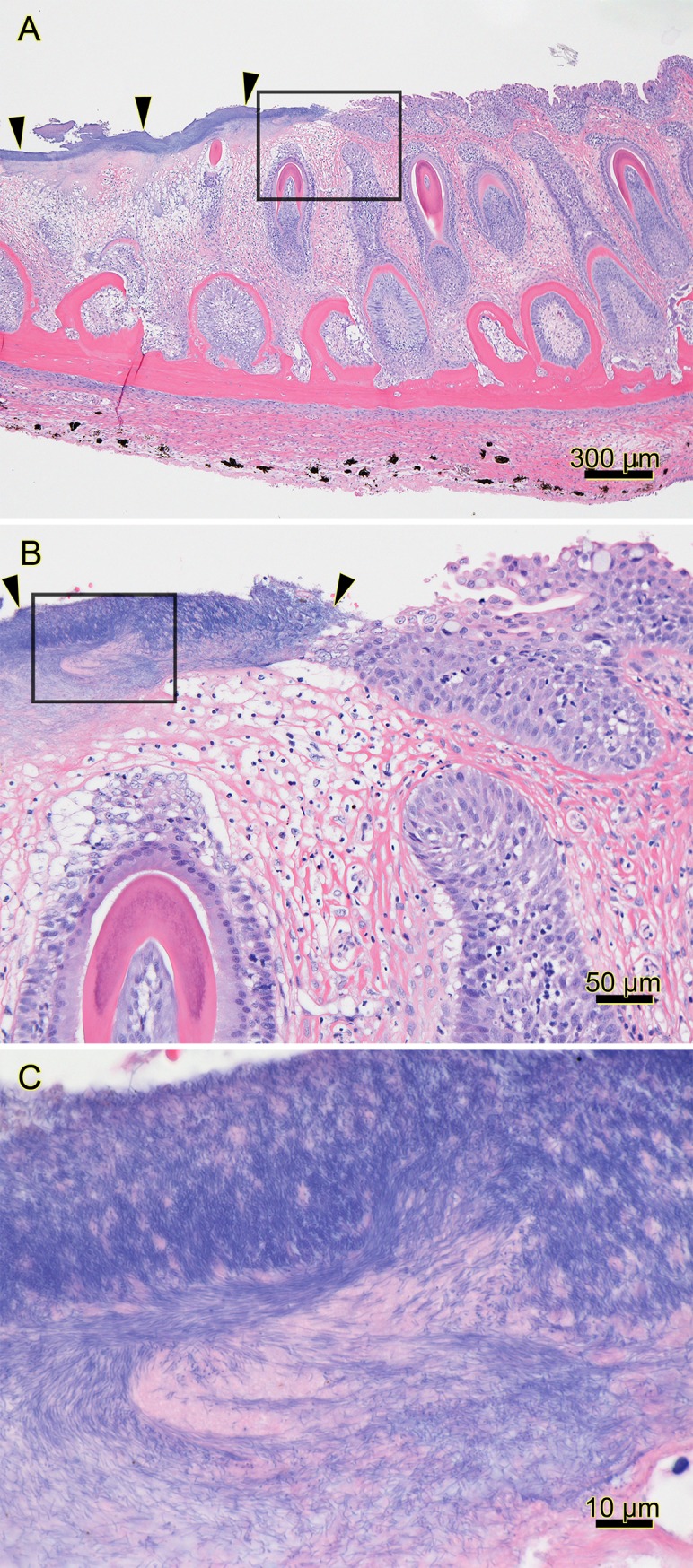
Histopathology of the jaw of a smolt from a natural outbreak of mouthrot. Histopathology of the jaw from a farmed Atlantic salmon that died 2 months after it was transferred from freshwater into a saltwater net-pen in BC; H&E stain. (A) The mucosal epithelium on the left side of the section is ulcerated and covered by a layer of deeply basophilic bacteria (arrowheads). The black box surrounds the transition from the bacteria-covered ulcer (left) to intact epithelium (right), and it outlines the area included in B. (B) Higher magnification of the transition between the ulcer covered by filamentous bacteria (arrowheads) and intact epithelium (right of right arrow); black box outlines the area included in C. (C) Higher magnification of abundant filamentous bacteria streaming in a proteinaceous matrix. (Optimization of photomicrograph illumination and color balance followed published methods [25]).

Representative tissues from the lesions (mouth, skin and gills) of diseased fish sampled from Atlantic salmon smolts bath infected with BC strains of *T*. *maritimum* [11] were fixed in 10% neutral buffered formalin solution and kept at 4°C until processing. The tissue processing and sectioning for histology were performed by a commercial laboratory. Histology sections were stained with hematoxylin and eosin (H&E). Histology sections from a diseased smolt from a natural outbreak of mouthrot at a BC farm were used as a reference (Fig 1).

Tissues (mouth and skin) from experimentally infected smolts were also selected for scanning electron microscopy (SEM) examination. Preparation of tissues for SEM was performed as described in Småge, Frisch [21].

## Results

### Real-time RT-PCR for *T*. *maritimum*

The Tmar_ompA assay is specific to *T*. *maritimum* based on the testing of RNA extracted from the *T*. *maritimum* strains (*T*. *maritimum* strains NCIMB 2154^T^, TmarCan15-1, TmarCan16-1, TmarCan16-5, NLF-15, and Ch-2402) and RNA extracted from other *Tenacibaculum* species (*T*. *adriaticum* DSM18961^T^, *T*. *dicentrarchi* USC35/09^T^, "*T*. *finnmarkense*" HFJ^T^, *T*. *ovolyticum* EKD-002^T^, and *T*. *soleae* LL0412.1.7^T^). When compared to assay developed by Fringuelli, Savage [19], Tmar_ompA is less sensitive (S1 Table). The efficiency of Tmar_ompA is 1.9138 for pure *T*. *maritimum* culture (TmarCan15-1) and 1.9386 for *T*. *maritimum* positive skin tissue (S1 Table).

### Cohabitation experiment tissue screening

All samples from diseased cohabitants were positive for *T*. *maritimum* using the newly developed Tmar_ompA assay (S2 Table). Bacterial loads were higher in the gills and mouth of the groups exposed to the two less pathogenic isolates (TmarCan15-1 and TmarCan16-5). Results from the heart, brain and kidney samples showed that *T*. *maritimum* was in all three of these tissues in clinically affected cohabitant fish, indicating that the bacteria or the detected segments become systemic. *T*. *maritimum* was also detected in most of the sampled tissues in the randomly sampled non-diseased cohabitants (S2 Table). Although a majority of these were positive, not all internal tissues were positive in all individuals. Cohabitants from the control groups were screened by Frisch, Småge [11] and were negative for *T*. *maritimum*.

### Clinical signs

**Fig 2:**
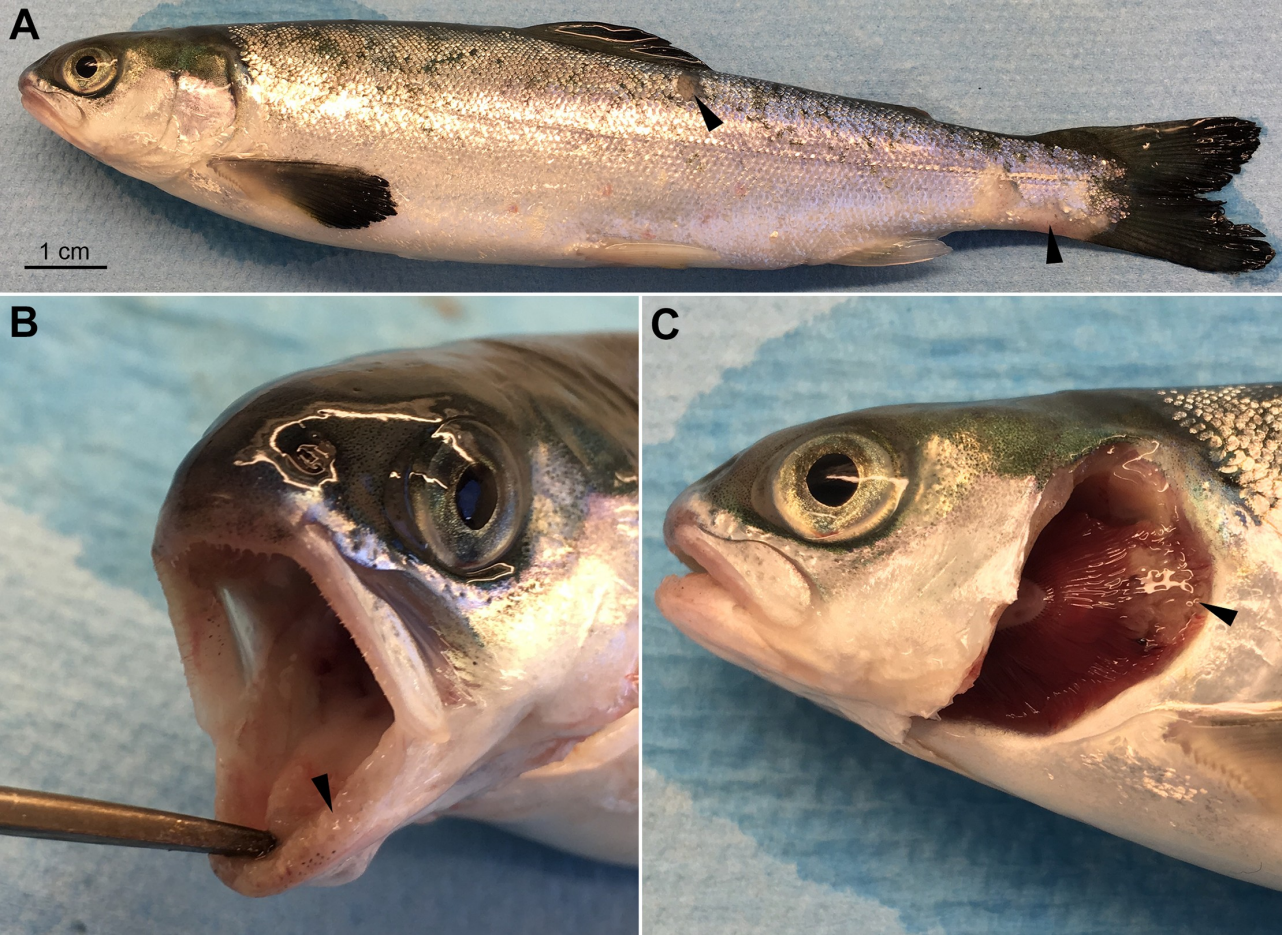
Gross clinical signs of an experimentally infected smolt. A moribund Atlantic salmon smolt that was bath infected with *T*. *maritimum* strain TmarCan16-1. Gross lesion scoring [11]: mouth = 2 out of 3, skin = 1 out of 3, gills = 1 out of 2. (**A**) Very few clinical signs are on the body surface other than some scale loss at the base of the peduncle and dorsal-lateral surface (arrows). (**B**) The gingiva is swollen (arrow). (**C**) A gill lesion (arrow).

As described in Frisch, Småge [11], Atlantic salmon smolts bath infected with *T*. *maritimum* strains from BC presented with very few external (Fig 2) or internal clinical signs. Mouth lesions were the most common finding, with some fish also having skin and/or gill lesions. Mouth lesions were usually on or surrounding the teeth and tongue (Fig 2B) and were associated with a slime layer that generally had a yellow tinge. This slime contained a large quantity of long thin rod-shaped bacteria with *T*. *maritimum* morphology [11]. When lesions were on the skin (Fig 2A) or gills (Fig 2C), these were also linked with a slime layer containing large amounts of bacteria with *T*. *maritimum* morphology.

### Microscopic pathology

**Fig 3:**
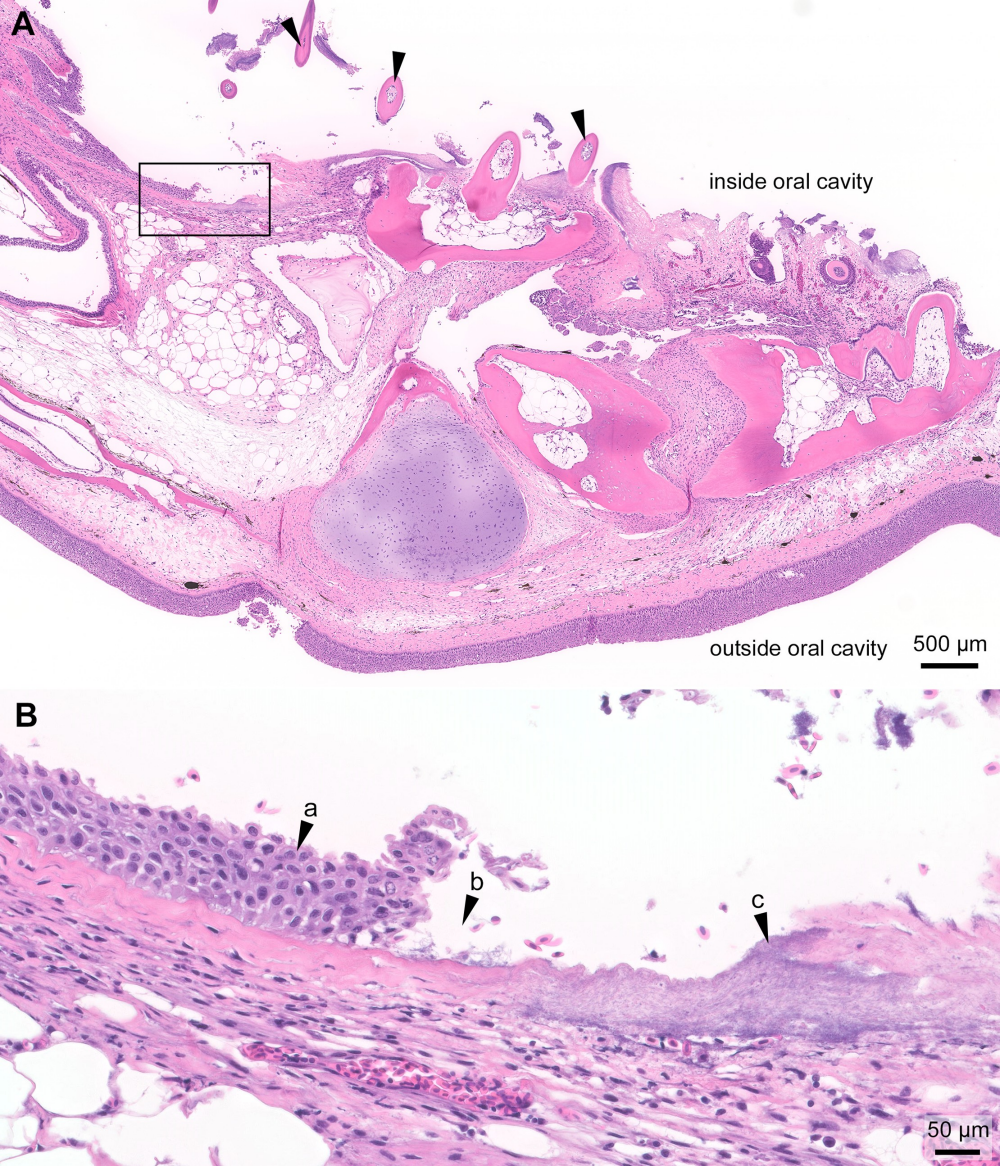
Histopathology of the jaw from an experimentally infected smolt. Histopathology of the gills from a moribund Atlantic salmon smolt experimentally bath infected with *T*. *maritimum* strain TmarCan15-1; H&E stain. (**A**) Oblique section of the jaw with mouthrot and loose teeth (arrowheads) with only a few of them connected to the jaw. The top is the inside of the oral cavity and the bottom the outside. The epidermis on the outside is intact. The black box outlines the area included in B and represents the transition at the edge of the ulcer. (**B**) The distance between intact mucosal epithelium (arrow "a") and the ulcer (arrow "b") is very short. Large quantities of bacteria with *T*. *maritimum* morphology are within the ulcer (arrow "c"). No signs of inflammation at the edge of the ulcer.

**Fig 4:**
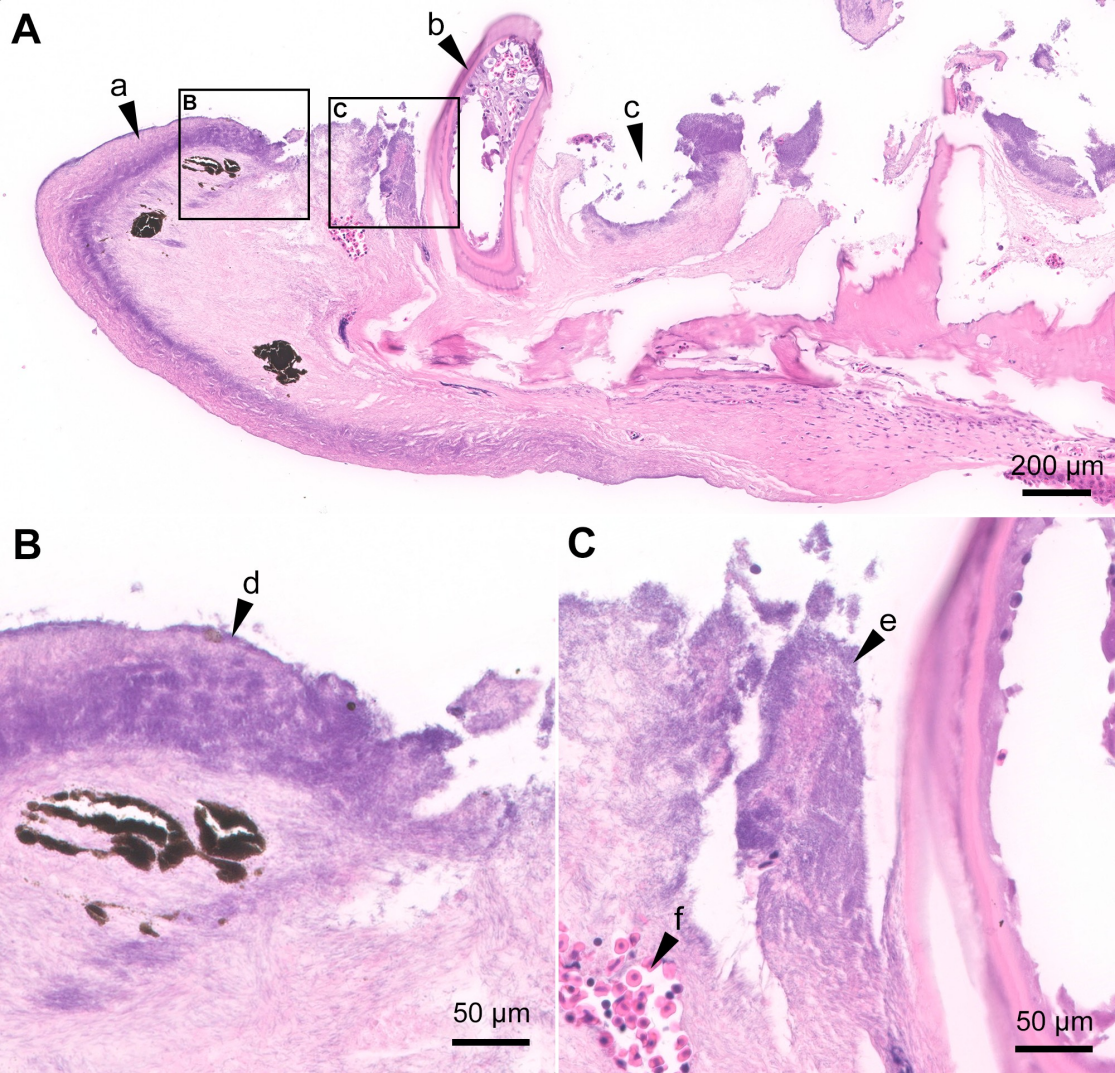
Histopathology of the jaw from an experimentally infected smolt. Histopathology of the jaw from the smolt in Fig 2; H&E stain. (**A**) Oblique section of the jaw. The epidermis is completely missing and the outer surface is covered with a thick mat of long thin rod-shaped *T*. *maritimum*-like bacteria that have infiltrated the submucosa (arrow "a"). Only one tooth (arrow "b") remains and holes are present where there used to be more teeth (arrow "c"). The black boxes labelled "B" and "C" outline the areas included in Fig 4B and 4C. (**B**) A mat of bacteria with *T*. *maritimum* morphology is on the outer surface (arrow "d") and the bacteria have infiltrated the underlying submucosa. (**C**) Large quantities of bacteria with *T*. *maritimum* morphology are within the destructed submucosa surrounding the tooth (arrow "e"). Some intact red blood cells (arrow "f") are within the mass of bacteria and remnants of tissue.

In the experimentally infected smolts, histopathological changes are mainly present in the mouth, and some fish have gill and/or skin lesions. Generally, these changes are associated with the gross lesions (Fig 2). The gross oral lesions (Fig 2B) are microscopically associated with mats of long thin rod-shaped bacteria matching what is described for *T*. *maritimum* (Figs 3 and 4). The severity of the histopathology varies between individuals. The distance between intact epidermis with no signs of structural damage to an open ulcer with large quantities of bacteria is very short (Fig 3). In most cases, little or no inflammation surrounds lesions (Fig 3B). Large quantities of bacteria with *T*. *maritimum* morphology are present in the gingival pockets surrounding the teeth and these are often loose and, in some cases, falling out or completely missing (Figs 3 and 4). In severe cases, normal tissue structures are replaced by a structureless mass of large amounts of bacteria and cellular debris (Fig 4).

**Fig 5:**
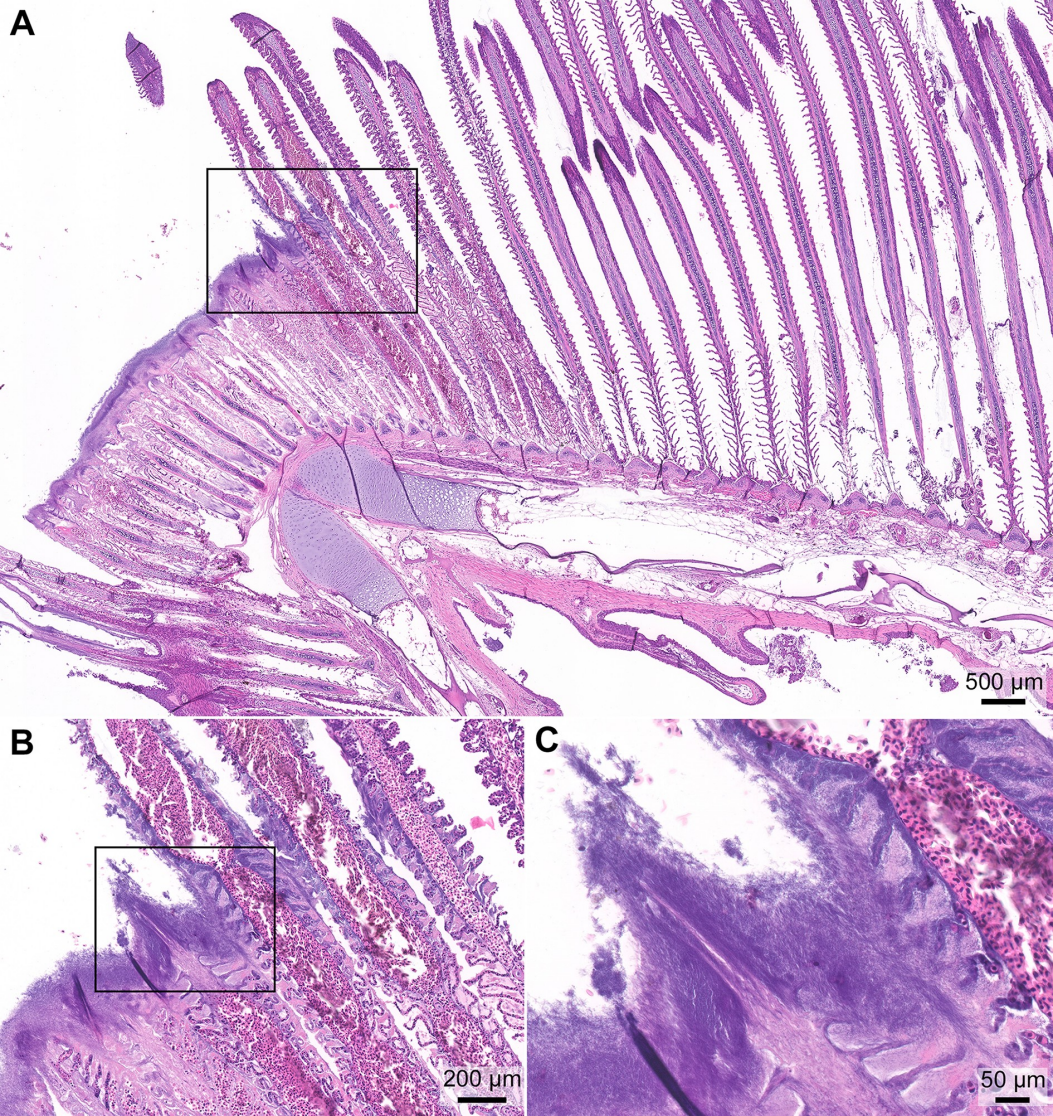
Histopathology of the gills from an experimentally infected smolt. Histopathology of the gills from the smolt in Fig 2; H&E stain. (**A**) Section of the gills with a distinct lesion on the top of the curve of the gill arch. The tips of the filaments are missing in the center of the lesion, and the remaining distal end of the filament is necrotic. The tissue is replaced by a thick layer of bacteria with *T*. *maritimum* morphology. The black box includes the transition between the lesion and normal tissue and outlines the area included in B. (**B**) The distance between the lesion and normal gill filaments is very short. In the damaged area, only the blood vessels remain in some of the lamellae. The black box outlines the area included in C. (**C**) Abundant bacteria with *T*. *maritimum* morphology cover the destroyed region of the gills. Only remnants of the lamellae are within the ulcer.

Most of the examined gills from the experimentally infected smolts have no microscopic changes associated with disease and were deemed “healthy”; however, gills with macroscopic lesions have significant microscopic changes (Fig 5). As with the mouth lesions, there is a total loss of cell and tissue structure linked to these lesions with little or no inflammation and large amounts of bacteria with *T*. *maritimum* morphology. Most of the gill lesions occurred at the curve of the gill arch (Figs 2C and 5A). The tip of the filaments in affected areas is completely destroyed and replaced by a thick layer of bacteria with *T*. *maritimum* morphology (Fig 5A). The distance between the ulcer and the intact filaments of the gills is very short (Fig 5A and 5B). Only remnants of the lamellae are within the ulcer (Fig 5B and 5C).

**Fig 6:**
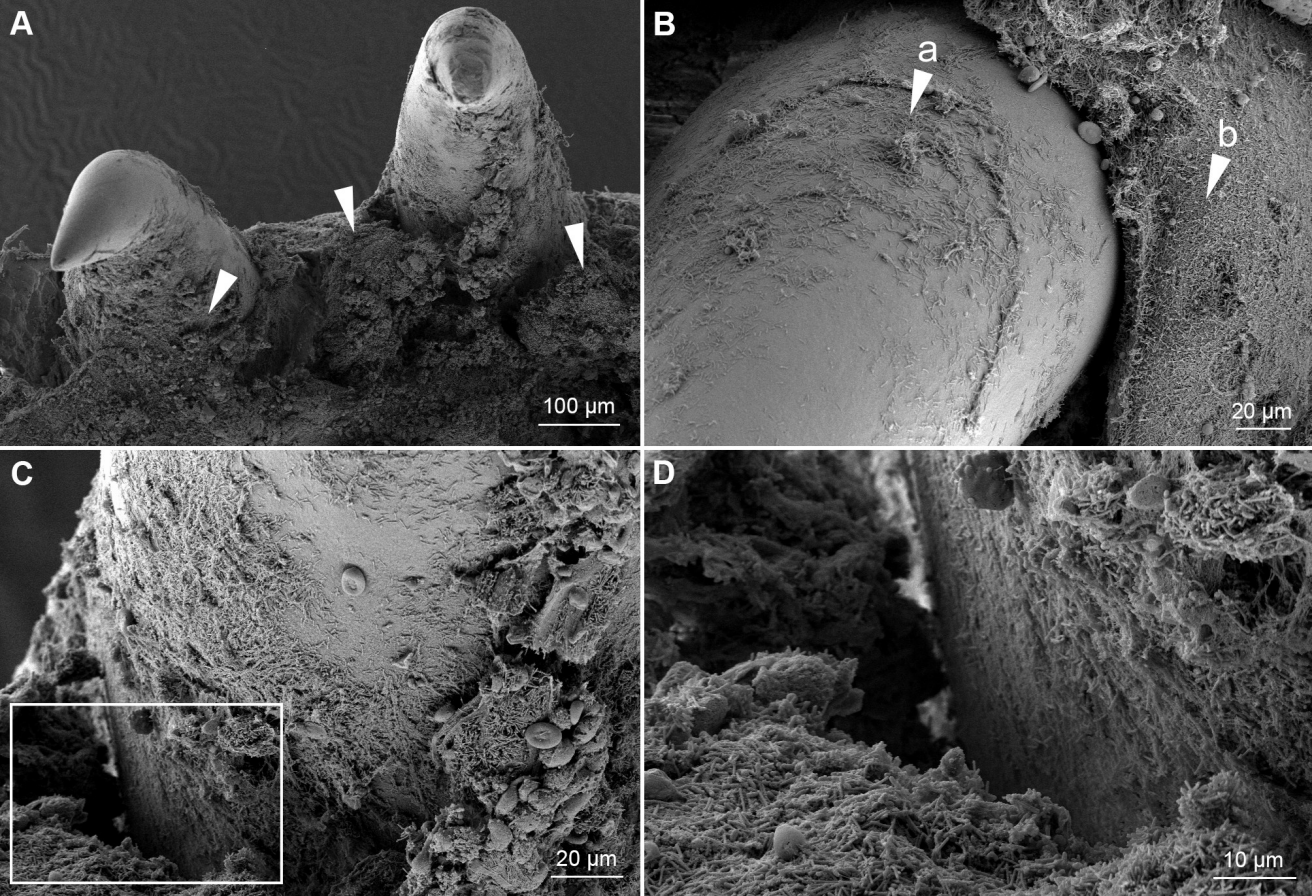
SEM of teeth from an experimentally infected smolt. Micrographs of teeth and the surrounding tissue from the mouth of a diseased smolt bath infected with TmarCan15-1 in the cohabitation experiment. (**A**) Teeth and surrounding gingiva are covered by mats of bacteria with *T*. *maritimum* morphology (arrowheads) and the associated tissue is damaged. (**B**) Zoomed in view of a tooth showing bacterial growth on the surface of the tooth (arrow "a") as well as the surrounding gingival tissue (arrow "b"). (**C**) The dentin-enameloid interface with associated tissue destruction. White box indicates area in D. (**D**) Cellular debris within the bacterial mats.

**Fig 7:**
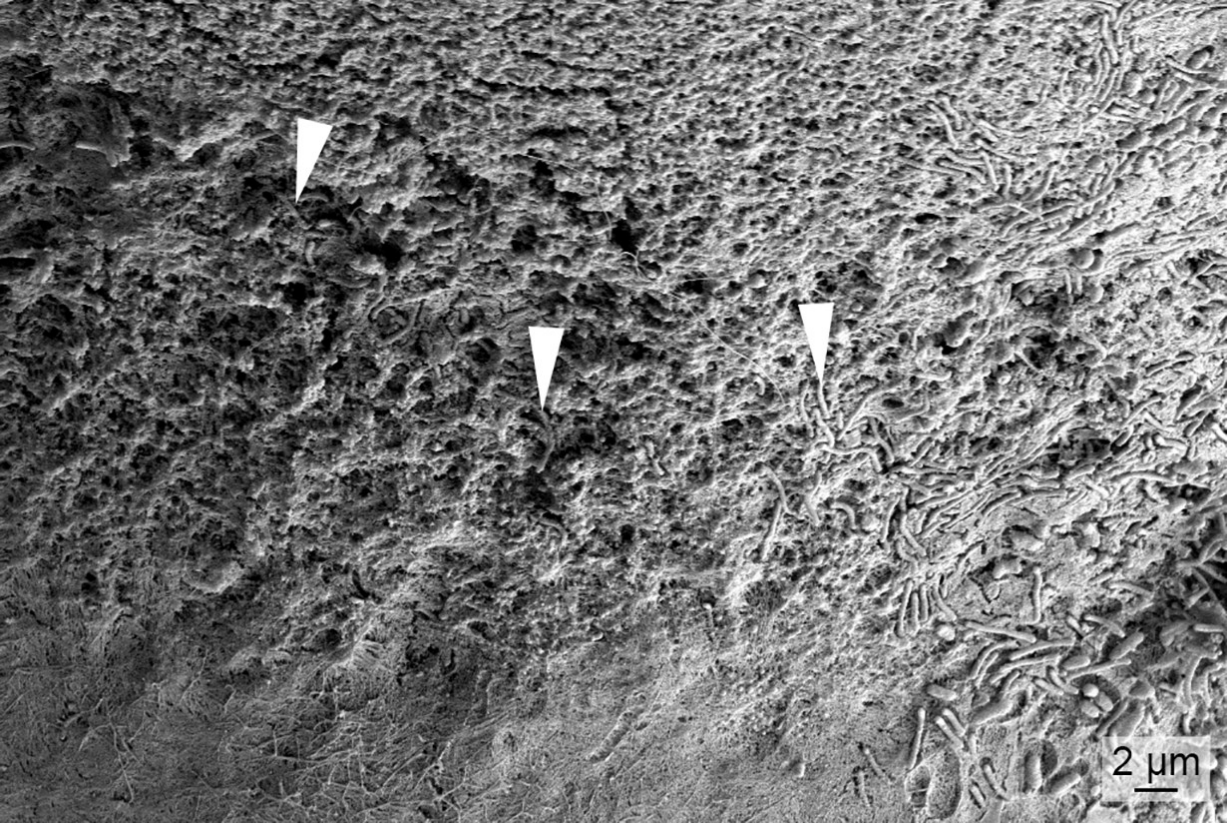
SEM of a tooth surface from an experimentally infected smolt. Micrograph of a tooth surface from a diseased smolt bath infected with TmarCan15-1 in the cohabitation experiment. Bacteria with *T*. *maritimum* morphology are within the enameloid of the tooth (arrowheads).

**Fig 8:**
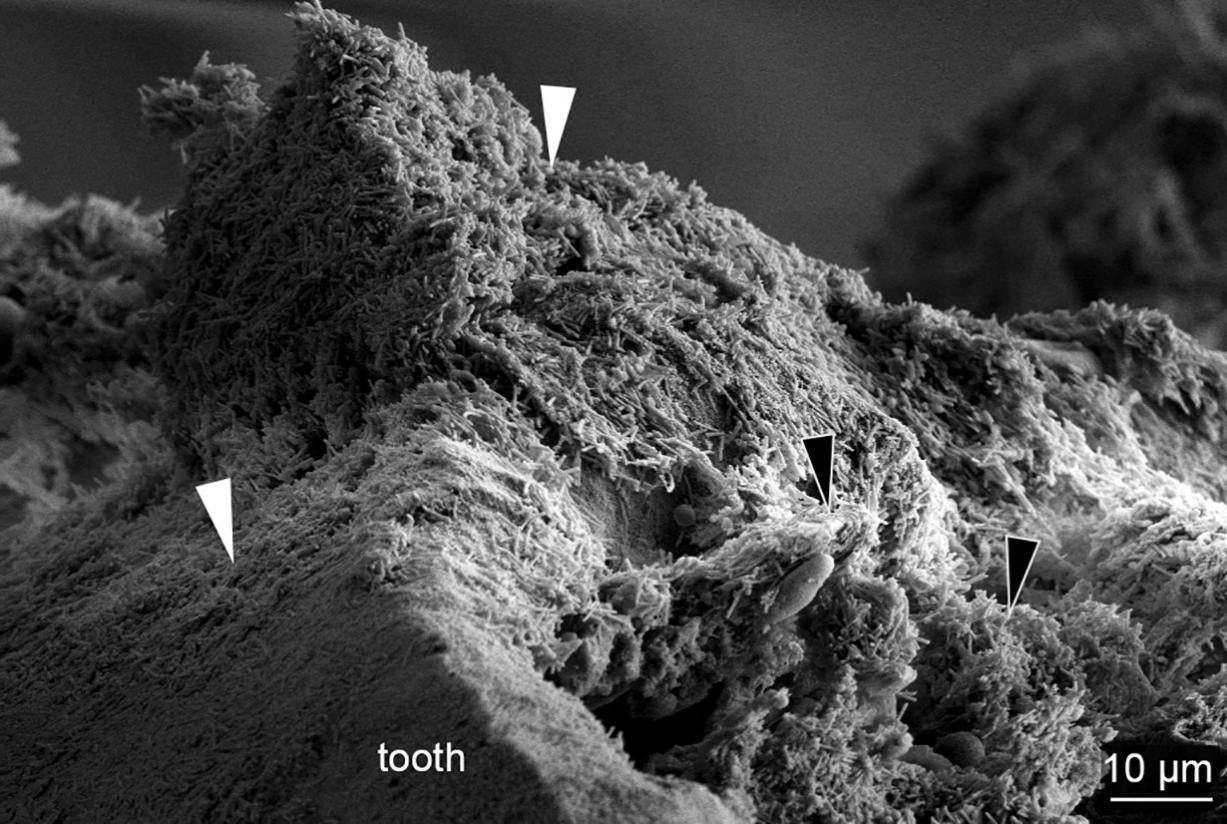
SEM of a fractured tooth from an experimentally infected smolt. Micrograph of a fractured tooth from a diseased smolt bath infected with TmarCan15-1 in the cohabitation experiment. Large aggregates of bacteria with *T*. *maritimum* morphology are on the outside of the tooth (white arrowheads) as well as within the exposed pulp (black arrowheads) of the tooth.

**Fig 9:**
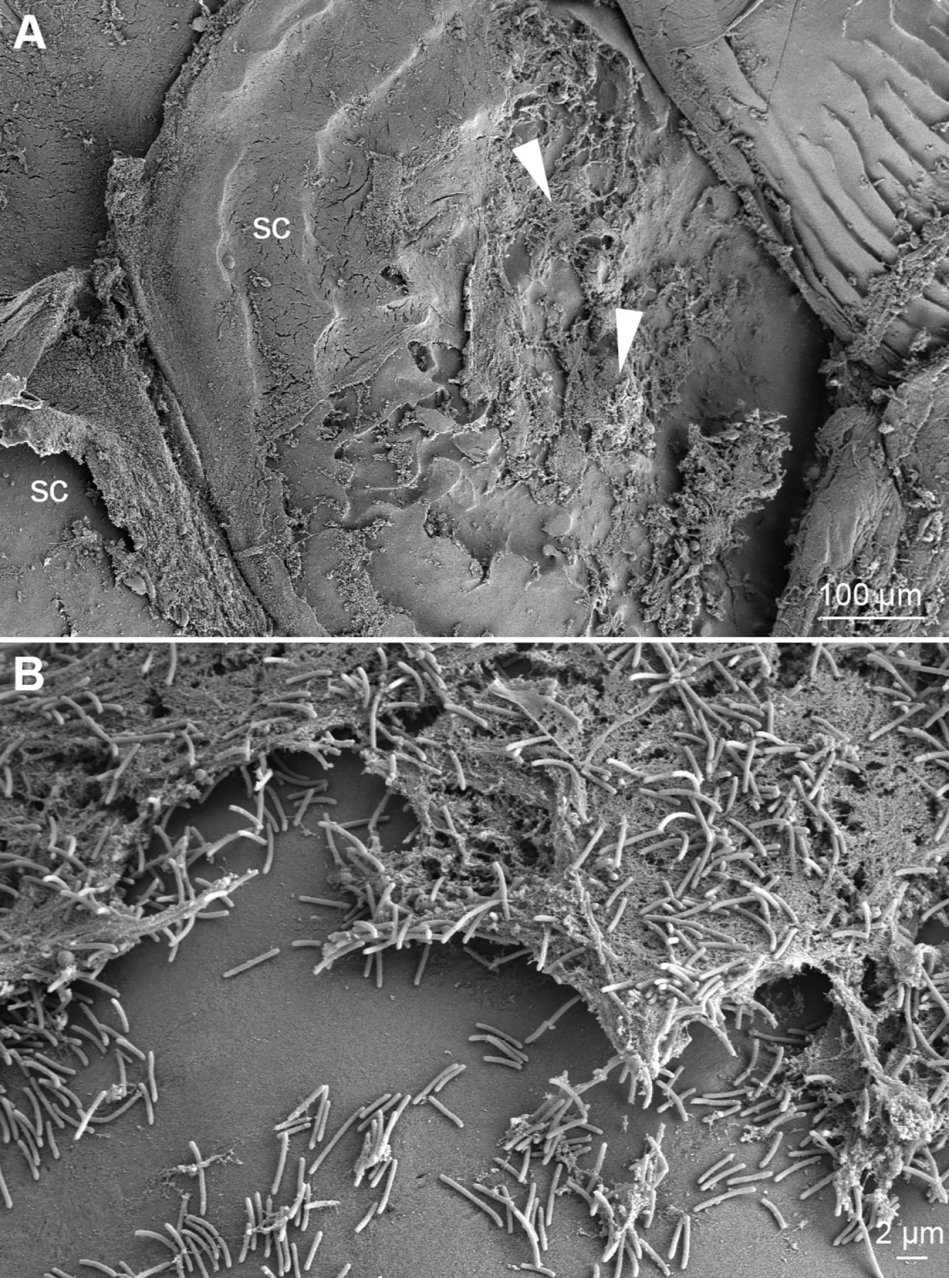
SEM of a skin lesion from an experimentally infected smolt. Micrographs of a skin lesion on the dorsal-lateral surface of a diseased smolt bath infected with TmarCan15-1 in the cohabitation experiment. (**A**) Mats of bacteria with *T*. *maritimum* morphology (arrowheads) are associated with epithelial damage exposing the scales (sc). (**B**) Cellular debris with aggregates of bacteria with *T*. *maritimum* morphology.

The skin lesions that developed during the experiments were associated with scale pocket edema. Total destruction of the underlying tissue is replaced with mats of bacteria with *T*. *maritimum* morphology. The SEM micrographs support the histopathological findings. Large aggregates of bacteria with *T*. *maritimum* morphology are in the areas of tissue destruction and surrounding the teeth (Fig 6). Cellular debris is clearly visible within these bacterial mats (Fig 6C and 6D). The bacteria are embedded in the surface of some of the teeth (Fig 7). Some teeth are fractured and bacterial aggregates are within the exposed pulp of these teeth (Fig 8). Bacterial mats and aggregates with associated tissue destruction are also in the skin lesions (Fig 9).

## Discussion

The macroscopic and microscopic findings of experimentally induced mouthrot described in this study match the pathology in field cases (Fig 1), as well as what is described in the literature [12,14]. Comparing our findings to publications is difficult as most of these were written in the 1980s and 1990s before the *Tenacibaculum* genus was described and it is therefore difficult to make a meaningful comparison. Bacterial mats with *T*. *maritimum* morphology typically surround the teeth, and bacterial cells are seen within the gingival epithelium invading the tissues below. This suggests that the bacteria proliferate in the gingival pockets surrounding the teeth and spread to the surrounding tissues as was described by Frelier, Elston [12]. The SEM micrographs (Fig 6) add to the picture by showing that the bacteria adhere to the tooth surface and epithelium, creating large aggregates. This is associated with destruction of the surrounding tissues.

Skin lesions with associated scale pocket edema that matched the description by Handlinger, Soltani [7] occurred in a subset of Western Canadian *T*. *maritimum* experimentally infected smolts, particularly ones with a more chronic presentation [11]. Skin lesions, which are not common in natural outbreaks of mouthrot, may be attributed to the use of tanks that result in a greater potential for physical skin abrasions than saltwater net-pens. The use of dip nets to transfer the smolts in and out of the challenge tanks may also have contributed to this by disrupting the protective mucus layer and causing scale loss. The greater prevalence of gill lesions in experimentally infected smolts might be due to the clumping nature of *T*. *maritimum* that may create bacterial aggregates capable of lodging themselves in the gill filaments during respiration. This hypothesis is supported by the finding in the cohabitation experiment that fewer cohabitants had gill lesions than the shedders that were directly exposed to the bacterial culture during the bath infection (Table 2) [11].

The reasons why *T*. *maritimum* targets the teeth and surrounding mucosa in mouthrot are not fully understood. However, the teeth are a high source of calcium that has been shown to promote the growth of *T*. *maritimum* [26] and thus may contribute to the affinity for this particular tissue. Also, a gene encoding a collagenase has been identified in the whole genome sequence of *T*. *maritimum* [27] and likely the reason why high levels of *T*. *maritimum* are present in the collagen-rich submucosa (Figs 3 and 4). *T*. *maritimum* is also strongly adhesive to hydrophobic surfaces, including fish mucus [28,29]. This ability to adhere and colonize is an important first step for pathogenic bacteria to invade the host [30]. This is likely the main mechanism by which *T*. *maritimum* is able to create biofilms so effectively. Biofilms, created by many pathogenic bacteria including *Staphylococcus aureus*, provide resistance against many host defense mechanisms [31], and may explain the low level of immune response in mouthrot.

We developed a new real-time RT-PCR assay based on the *ompA* gene that is as specific but less sensitive than the published assay based on the 16S rRNA gene [19]. The results from the real-time RT-PCR tissue screening performed in this study and the recovery of the bacteria from kidneys of experimentally diseased fish [11] provide evidence that mouthrot is a systemic disease. However, no significant pathology occurred in internal organs [11]. This is further supported by the fact that when examining mouthrot affected smolts from the field, lesions in other organs are not obviously associated with mouthrot but further research is required to determine if such a link exists (personal communication, Gary Marty). The microscopic pathology of the mouth suggests that *T*. *maritimum* might be entering the highly vascular tooth pulp (Fig 8) once significant damage has occurred to the tooth and surround tissues. This may provide an entry point to the bloodstream, to then become systemic. This hypothesis matches what is described for periodontal disease in mammals. The lack of visible internal pathology, as well as the lack of observable inflammatory response may reflect the acuteness of the disease and resulting rapid tissue destruction. This is likely due to toxins with high proteolytic activity produced by *T*. *maritimum* [7,27,32–34].

The real-time RT-PCR screening of the cohabitants showed that the external tissues (gills, mouth and mucus) of the fish infected with TmarCan16-1 had a lower load of *T*. *maritimum* than TmarCan15-1 and TmarCan16-5. This is interesting in view of the fact that this isolate results in a more rapid and severe disease (Table 1) with less severe gross clinical signs (Table 2). This relationship between highly pathogenic strains and a lack of severe lesions has previously been noted before for flavobacteria [30]. The real-time RT-PCR results are therefore not an indicator of pathogenicity. Variation in pathogenicity between *T*. *maritimum* strains has been shown in other studies, including other fish species [2,35,36]. Differences in pathogenicity also occur between isolates belonging to the same multilocus sequence type (genetically identical on 11 housekeeping gene sequences) as was the case for TmarCan16-1 and TmarCan16-2 [11]. Further analysis of the genome of TmarCan16-1 and TmarCan16-2 is required to identify the potential differences in virulence factors resulting in the observed variation in pathogenicity.

The pathology in this study is different to what has been described in both experimentally and naturally infected farmed Atlantic salmon smolts in Tasmania, Australia with *T*. *maritimum* [7]. In Tasmania, the pathology has to a greater extent resembled what is described for typical tenacibaculosis: frayed fins, tail rot, skin lesions/ulcer and mouth erosion [7,17]. The reason behind these pathological differences is not known. It could be due to a difference in the *T*. *maritimum* strains associated with the different pathological presentations, but it could be due to other factors, including host and environment. One possibility is that the experiments were conducted at different temperatures, 12°C in our study and around 18–20°C in the experiments in Tasmania [7,17,36,37]. Pathogenicity differences associated with temperature has been shown *in vitro* with *M*. *viscosa*, a different skin pathogen of Atlantic salmon [38].

## Conclusion

The mechanism by which *T*. *maritimum* kills smolts in the Pacific Northwest still remains a mystery. The main pathology in experimentally infected smolts with Western Canadian *T*. *maritimum* strains are mouth lesions that damage the tissues surrounding the teeth causing a disease that is similar to periodontal disease in mammals. The pathological changes are focal, severe, and occur very rapidly with very little associated inflammation. *T*. *maritimum* is detectable internally by real-time RT-PCR and bacteriology, and one possible point of entry would be the teeth.

## Supporting information

S1 Table Real-time RT-PCR results for the development of the Tmar_ompA assay. Ct values of the specificity and efficiency analyses performed during the development of the Tmar_ompA assay.
(XLSX)

S2 Table Cohabitation experiment real-time RT-PCR results. Ct values of the real-time RT-PCR analysis performed on diseased and non-diseased cohabitants.
(XLSX)
